# *β*-Cyfluthrin-Mediated Cytotoxicity of Cultured Rat Primary Hepatocytes Ameliorated by Cotreatment with Luteolin

**DOI:** 10.1155/2022/3647988

**Published:** 2022-08-27

**Authors:** Lachin Mousavi, Elham Zadeh-Hashem, Mehdi Imani

**Affiliations:** Department of Basic Sciences, Faculty of Veterinary Medicine, Urmia University, Urmia, Iran

## Abstract

The current study was designed to evaluate the possible protective effects of luteolin against *β*-cyfluthrin-mediated toxicity on the primary culture of rat hepatocytes (RHs). In the first step, the exposure of RHs to *β*-cyfluthrin (10, 20, 40, and 80 *μ*M) was assessed by MTT. Second, redox condition was evaluated in cotreatment of cells with luteolin (20, 40, and 60 *μ*M) and *β*-cyfluthrin (40 *μ*M) at both medium and intra levels. In comparison to control, viability was lower in 40 and 80 *μ*M *β*-cyfluthrin-treated groups at 24 h and all *β*-cyfluthrin-treated groups at 48 h (*P* < 0.05). Cotreatment with 20 or 40 *μ*M luteolin + 40 *μ*M *β*-cyfluthrin resulted in a higher viability value compared to *β*-cyfluthrin alone at 24 and 48 h of incubation (*P* < 0.05). Administration of 20 or 40 *μ*M luteolin with *β*-cyfluthrin led to the decrease of malondialdehyde and total nitrate/nitrite and the increase of total antioxidant capacity (TAC) values in both medium and intrahepatocyte levels compared to the *β*-cyfluthrin-treated group at 48 h (*P* < 0.05). It seems that low and medium doses of luteolin possess the potential to reduce *β*-cyfluthrin-mediated hepatotoxicity via attenuation of peroxidative/nitrosative reactions and augmentation of TAC levels.

## 1. Introduction

Pesticides are used to control pests widespread. However, they could induce toxicity in nontarget species, such as animals and humans, inadvertently [1]. Since the last few decades, pyrethroids have been the most widely used class of pesticides worldwide [2]. Due to the relative safety of *β*-cyfluthrin, the assorted type II pyrethroid pesticide, it is extensively used to control vermin [3]. It induces persistent membrane depolarization of the nervous system due to the impairment of sodium-ion-gated channels. Axon sodium channels of mammals are significantly less sensitive to the toxic effects of pyrethroids than that of insects. However, the toxicity of *β*-cyfluthrin on different tissues and organs has been reported by researchers [4–9]. In this regard, oxidative and nitrosative effects of *β*-cyfluthrin during *in vitro* preservation of spermatozoa were also indicated [10]. Moreover, the stimulatory effect of *β*-cyfluthrin on the generation of reactive oxygen species (ROS) and ultimately related oxidative toxicity has been demonstrated in rat kidneys, aquatic organisms, and erythrocytes of rabbits [11–13]. Studies indicated that exposure to pyrethroid resulted in oxidative toxicity, immunological and sex hormone disturbances, gene mutations, damages to DNA of spermatozoa, and a decrease in semen quality [4, 7]. Therefore, the knowledge about redox balance is an essential aspect of research in humans and animals.

Luteolin (3, 4, 5, 7-tetrahydroxylflavone) is a flavonoid compound found in many fruits and vegetables [14]. Antineoplastic, antihepatotoxic, antiallergic, antioxidative, antiadipogenic, and anti-inflammatory effects of luteolin were demonstrated by several research studies [15–20]. It has been reported that luteolin was able to reduce the production of ROS, modulate the redox balance, and ultimately reduce the adverse effects of peroxidative reactions in biological systems [21]. Moreover, research displayed that luteolin ameliorated the adverse effect of glucocorticoid by increasing gene and protein expression of hepatic and renal enzymes [22]. The conventional protective mechanisms of luteolin against induced hepatotoxicity by acetaminophen or tetrachloromethane are the upregulation of enzymatic antioxidants and alleviation of proinflammatory mediator's expression [23, 24]. Recently, research projects have focused on preventing or curing diseases mediated by environmental toxicants using naturally derived compounds [25, 26]. However, the role and the possible mechanism of luteolin against *β*-cyfluthrin-mediated hepatotoxicity have not been indicated. We speculated that the antioxidative role of luteolin would neutralize the ROS production and peroxidative effects of *β*-cyfluthrin on hepatocytes. Thus, the first objective of the current study was to find the mild-moderate toxic doses of *β*-cyfluthrin during the primary culture of rat hepatocytes. Then, the protective effect of luteolin by coadministration of determined toxic doses of *β*-cyfluthrin was assessed, as well.

## 2. Materials and Methods

### 2.1. Experimental Rats and Isolation of Hepatic Cells

The rats were kept in controlled environmental houses. The Animal Care Committee of Urmia University approved the procedure of study (ethical approval number: IR-UU-AEC-3/PD/32). Adult male Wistar rats (200–250 g; *n* = 35) were anesthetized with chloroform. Hepatocytes were isolated according to the method described previously [27]. Briefly, an angiocath (needle gauge = 24) was gently inserted into the portal vein, and the liver was perfused using CMF-HBSS solution (pH = 7) for 10 min (flow rate of 10 mL per min). The flushed perfusate was collected via the inferior vena cava and discarded. Following the flushing of the circulating blood from the liver, the second step of perfusion was performed with CMF-HBSS containing trypsin 0.1% (T4799; Sigma-Aldrich, Chemie GmbH, Germany) for 30 min. Then, the minced liver tissue was pipetted up and down in cold CMF-HBSS. Then, the cell suspension was filtered through a mesh (size: 150 *μ*m) and then centrifuged for 15 min at 500 rpm. The pellet was resuspended in 30 mL of CMF-HBSS and recentrifuged (repeated three times). Finally, the recovered hepatocytes were suspended in Dulbecco's modified Eagle medium (DMEM, P04-05551; PAN-Biotech, Germany) supplemented with penicillin G sodium/streptomycin sulfate (Solarbio® Life Science, Beijing, China) and 10% (v/v) heat-inactivated fetal bovine serum at a density of 200,000 cells/mL (counted using a hemocytometer). The viability of hepatocytes over 80% (assessed by the trypan blue exclusion test) was approved for the subsequent experiment. Hepatocytes were seeded onto 24-well plates (JET BIOFIL Tissue Culture Plate, 011024, China) and incubated at 37°C under 5% CO_2_/95% O_2_ overnight. Following preincubation, the medium and nonadherent hepatocytes were aspirated and replaced with fresh culture medium.

### 2.2. Experiment 1

In the first experiment, hepatocytes (2 × 10^5^ cells/well) were exposed to 0, 10, 20, 40, and 80 *μ*M concentrations of *β*-cyfluthrin (PESTANAL, 46003; Sigma-Aldrich, St Louis, MO) dissolved in dimethyl sulfoxide (DMSO; CARLO ERBA, France). Hepatocyte viability was assessed using 5-diphenyltetrazolium bromide (MTT) at 0, 24, and 48 h after exposure. The different stock solutions of *β*-cyfluthrin (80, 40, 20, 10, and 0 mM) dissolved in DMSO were prepared [10], and every well received an equal volume of DMSO-dissolved *β*-cyfluthrin (2 *μ*L in 1000 *μ*L medium) with different concentrations of *β*-cyfluthrin. A diluted sample was set as a negative control group at each run. The experiment was replicated for 6 times, and three wells were used for each group within each replication (number of biological replicates = 270).

### 2.3. Experiment 2

According to the MTT assay results of the first experiment, the dose of 40 *μ*M *β*-cyfluthrin was chosen for evaluation in the following experiment. In the second experiment, hepatocytes were exposed to *β*-cyfluthrin (40 *μ*M) alone and coadministered with 20, 40, and 60 *μ*M luteolin (72511, CAS No.: 491-70-3; Sigma-Aldrich, USA) as well. The stock solution of 10, 20, and 30 mM luteolin were prepared by dissolving an appropriate amount in the DMSO. The amount of DMSO in treated samples was less than 0.5% of the final volume. The viability of hepatocytes was assessed using MTT assay after 0, 24, and 48 h treatment. Moreover, malondialdehyde (MDA), total antioxidant capacity (TAC), total nitrite-nitrate (TNN), total lipid hydroperoxides (TLHPs), and superoxide dismutase (SOD) activity were evaluated in the medium and within hepatocytes at 0, 24, and 48 h after incubation, separately. The experiment was replicated for 8 times, and three wells were used for each group within each replication (number of biological replicates = 360).

### 2.4. Cell Viability Analysis

The viability of hepatocytes was evaluated by MTT assay. After treatment of hepatocytes, 10 *μ*L MTT (5 mg/mL in PBS) was added to each well and incubated for more four hours. The medium was aspirated, and DMSO was added to dissolve the formazan crystals. After 0.5–1 hour, the optical density of each well was read on a microplate reader (DANA, Iran) at the wavelength of 570 nm. Hepatocytes in the untreated control group were considered 100% viable, and then, viability in each tested group was expressed as a percentage of the untreated control group.

### 2.5. Measurement of Oxidative and Nitrosative Stress Indices and Antioxidant Levels in the Medium and Intrahepatocyte Homogenate Samples

The culture media were aspirated and collected within a tube. Then, each well was treated with 250 *μ*L trypsin-EDTA solution (0.25 and 0.02 g/100 mL, respectively) and placed for 10 min in an incubator. Separated hepatocytes were collected (within a new tube), and lysis solution (trichloroacetic acid 2.5%, 250 *μ*L) was added to the hepatocytes. In order to ensure the lysis of hepatocytes, homogenization was done using a cell homogenizer (T10 Basic; IKA®-Werke GmbH & Co. KG, Staufen, Germany) for 2 min. Then, the collected samples of culture media and hepatocyte suspensions were stored at freeze temperature (−20°C) for biochemical assay.

Malondialdehyde levels were assessed using the preparation of reagent described by Stern et al. [28]. After the mixing of TBA reagent with samples (culture media or homogenate of hepatocytes), incubation was performed (100°C, 15 min), and the tubes were centrifuged at 2500*g* for 15 min. The absorbance of the supernatant was recorded using a visible spectrophotometer (532 nm, Pharmacia NOVASPEC II; Pharmacia LKB). Concentrations of MDA were expressed as *μ*mol/g protein.

Solution and reagents required for the evaluation of TAC levels were prepared according to Koracevic and Koracevic [29]. After two steps of incubation, the absorbance of the generated color was recorded using a spectrophotometer (Pharmacia NOVASPEC II; Pharmacia LKB) at the wavelength of 532 nm against the blank solution. The amount of TAC was expressed as mmol/g protein in the homogenate of hepatocytes and cultured media.

Freshly prepared Griess reagent was used to measure the quantitative amount of TNN in the samples [30]. Following the mixing of reagent with the sample, the plate was incubated in darkness for 15 min at laboratory temperature, and the optical density of the generated solution color was recorded using an ELISA reader (DANA, Iran) at the wavelength of 540 nm. The amount of TNN was expressed as *μ*mol/g protein.

In order to measure SOD activity, pyrogallol and Tris buffer solutions were prepared freshly [31]. Finally, changes in the absorbance of samples (420 nm, Pharmacia NOVASPEC II; Pharmacia LKB) were compared with that of the control sample, and the SOD activity was calculated and expressed as unit/mg protein.

In order to evaluate the TLHPs, the working reagent, as described by Nourooz-Zadeh et al. [32], was prepared by dissolving recommended amounts of FOX-2 reagent with beta-hydroxytoluene. After mixing of the sample with a working solution, the tubes were incubated at the laboratory (dark place) for 30 min. Following completion of the reaction, the absorbance of the solution was recorded using a spectrophotometer (Pharmacia NOVASPEC II; Pharmacia LKB) at the wavelength of 560 nm. The standard curve was drawn using the absorbance of serial concentrations of hydrogen peroxide. The amount of TLHPs was expressed as *μ*mol/g protein.

### 2.6. Statistical Analysis

The interaction of time × treatment on different variables (MTT, MDA, TAC, TLHPs, TNN, SOD activity) among experimental groups was analyzed using two-way ANOVA, followed by a Holm–Sidak post hoc test. Results were presented as the mean ± S.E.M. The relationship between MTT and other variables (MDA, TAC, TNN, SOD, and TLHPs) were assessed by regression analysis. Statistical analyses were carried out using SigmaStat software (Version 3.5; Chicago, IL). Probability values lesser than 0.05 were considered significant.

## 3. Results

### 3.1. Experiment 1

**Figure 1:**
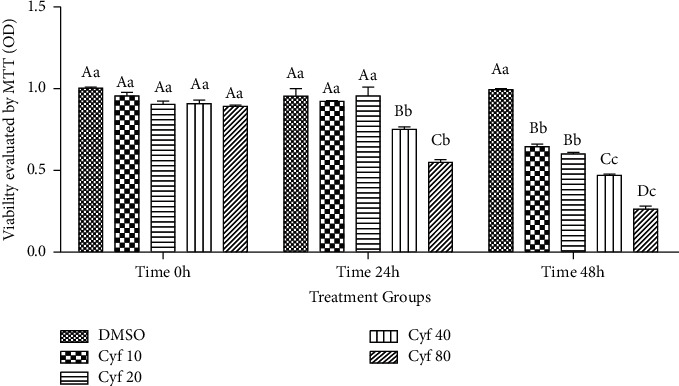
Effect of varying doses of *β*-cyfluthrin (10, 20, 40, and 80 *μ*M) on viability of hepatocytes (assessed by MTT) at different time point storages. Hepatocytes were seeded at 2 × 10^5^ cells/well and were incubated for 48 h. Data are expressed as the mean ± SEM. Hepatocyte viability was expressed and presented compared with the untreated hepatocyte control group. ^A,B,C,D^Values with different letters indicate a difference (*P* < 0.001) among groups at each time point. ^a,b,c^Values with different letters indicate a difference (*P* < 0.001) over time within every experimental group. Cyf, *β*-cyfluthrin; DMSO, dimethyl sulfoxide as a solvent of *β*-cyfluthrin.

Results of the first experiment revealed that viability (or functionality) of hepatocytes was not affected by *β*-cyfluthrin addition at 0 h (*P* > 0.05; Figure 1), while the mentioned index was reduced by exposure to 40 and 80 *μ*M *β*-cyfluthrin at 24 h and by all doses of *β*-cyfluthrin at 48 h of storage compared to the control group (*P* < 0.001; Figure 1). Finally, the 40 *μ*M *β*-cyfluthrin was considered as a mild-moderate toxic dose and hence selected for the subsequent experiments.

### 3.2. Experiment 2

#### 3.2.1. MTT

**Figure 2:**
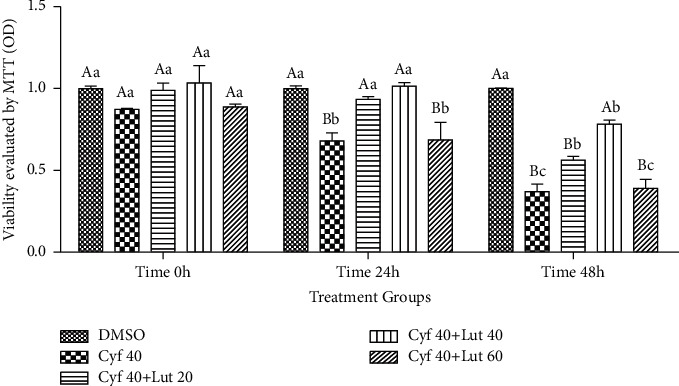
Effect of simultaneous treatment of luteolin (20, 40, and 60 *μ*M) with toxic doses of *β*-cyfluthrin (40 *μ*M) on viability of hepatocytes (assessed by MTT) at different time point storages. Hepatocytes were seeded at 2 × 10^5^ cells/well and were incubated for 48 h. Data are expressed as the mean ± SEM. Hepatocyte viability was expressed and presented compared with the untreated hepatocyte control group. ^A,B^Values with different letters indicate a difference (*P* < 0.05) among groups at each time point. ^a,b,c^Values with different letters indicate a difference (*P* < 0.05) over time within every experimental group. Cyf, *β*-cyfluthrin; Lut, luteolin; DMSO, dimethyl sulfoxide as a solvent of *β*-cyfluthrin and luteolin.

As shown in Figure 2, *β*-cyfluthrin (at 40 *μ*M) reduced the number of viable hepatocytes at 24 and 48 h time points. Cotreatment of luteolin (at 40 *μ*M) with toxic doses of *β*-cyfluthrin increased the viability of hepatocytes at mentioned time points (*P*=0.014). Within-group analysis indicated that viability was lower in *β*-cyfluthrin-treated groups (alone or cotreated with luteolin) at 48 h compared to 0 h (*P*=0.014; Figure 2).

#### 3.2.2. MDA (*μ*mol/g Protein) in the Cultured Media

**Figure 3:**
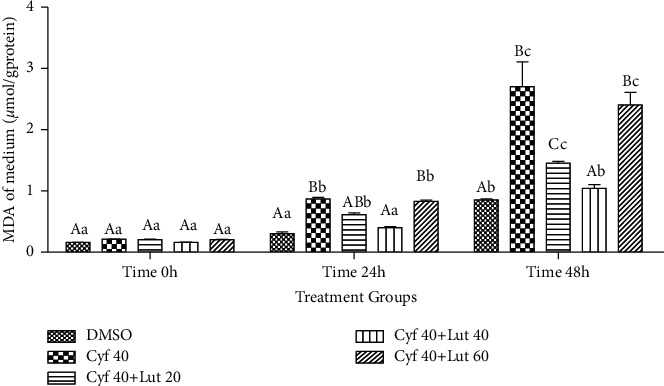
Amount of malondialdehyde (MDA; *μ*mol/g protein) in the cultured medium of rat hepatocytes following exposure to toxic doses of *β*-cyfluthrin (40 *μ*M) alone or cotreatment with luteolin (20, 40, and 60 *μ*M). Hepatocytes were seeded at 2 × 10^5^ cells/well and were incubated for 48 h. Data are expressed as the mean ± SEM. ^A,B,C^Values with different letters indicate a significant difference (*P* < 0.001) among groups at each time point. ^a,b,c^Values with different letters indicate a difference (*P* < 0.001) over time within every experimental group. Cyf, *β*-cyfluthrin; Lut, luteolin; DMSO, dimethyl sulfoxide as a solvent of *β*-cyfluthrin and luteolin.

The content of MDA, as a peroxidative variable, was not affected by treatment at 0 h (*P* > 0.05), while its amount was increased in *β*-cyfluthrin alone and *β*-cyfluthrin + 60 *μ*M luteolin-treated groups compared to the control group at other studied time points (*P* < 0.001; Figure 3). Luteolin at 40 *μ*M levels was able to reduce the deleterious effect of *β*-cyfluthrin on the peroxidative index at 24 and 48 h (*P* < 0.001; Figure 3). Changes over time revealed that the amount of MDA was greater at 48 h compared to 0 h in all experimental groups (*P* < 0.001; Figure 3).

#### 3.2.3. Intrahepatocyte Amount of MDA (*μ*mol/g Protein)

**Figure 4:**
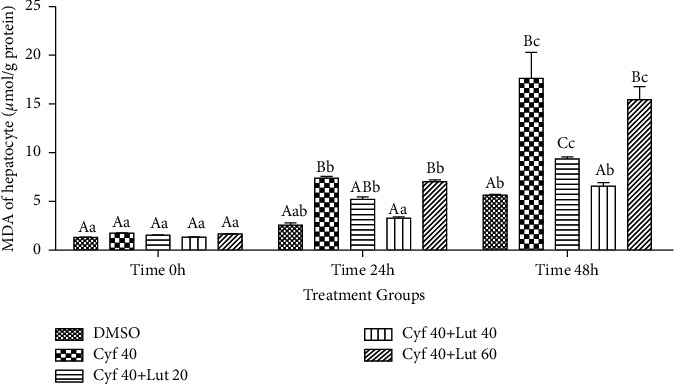
Amount of intrahepatocyte malondialdehyde (MDA; *μ*mol/g protein) following exposure to toxic doses of *β*-cyfluthrin (40 *μ*M) alone or cotreatment with luteolin (20, 40, and 60 *μ*M). Hepatocytes were seeded at 2 × 10^5^ cells/well and were incubated for 48 h. Data are expressed as the mean ± SEM. ^A,B,C^Values with different letters indicate a difference (*P* < 0.001) among groups at each time point. ^a,b,c^Values with different letters indicate a difference (*P* < 0.001) over time within every experimental group. Cyf, *β*-cyfluthrin; Lut, luteolin; DMSO, dimethyl sulfoxide as a solvent of *β*-cyfluthrin and luteolin.

The amount of MDA within the homogenate of hepatocytes was as same as in the cultured media. Neutralization of the adverse effect of *β*-cyfluthrin on the amount of intrahepatocyte MDA was achieved by 40 *μ*M luteolin (*P* < 0.001; Figure 4). Within-group analysis indicated that the amount of MDA was greater at 48 h compared to 0 h in all experimental groups (*P* < 0.001; Figure 4).

#### 3.2.4. TAC Levels (mmol/g Protein) in the Cultured Media

**Figure 5:**
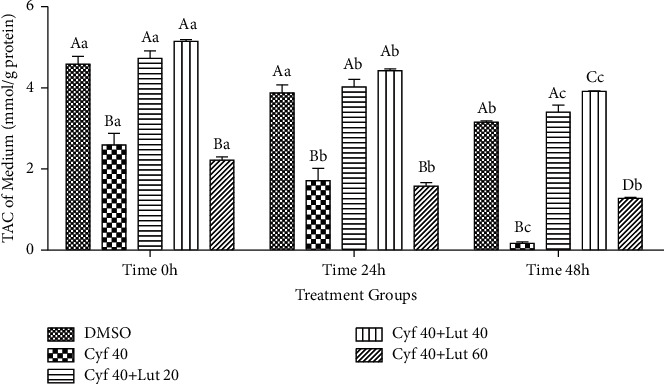
Total antioxidant capacity (TAC; mmol/g protein) in the cultured medium of rat hepatocytes following exposure to toxic doses of *β*-cyfluthrin (40 *μ*M) alone or cotreatment with luteolin (20, 40, and 60 *μ*M). Hepatocytes were seeded at 2 × 10^5^ cells/well and were incubated for 48 h. Data are expressed as the mean ± SEM. ^A,B,C,D^Values with different letters indicate a significant difference (*P* < 0.001) among groups at each time point. ^a,b,c^Values with different letters indicate a difference (*P* < 0.001) over time within every experimental group. Cyf, *β*-cyfluthrin; Lut, luteolin; DMSO, dimethyl sulfoxide as a solvent of *β*-cyfluthrin and luteolin.

Treatment with *β*-cyfluthrin alone or *β*-cyfluthrin + 60 *μ*M luteolin reduced the TAC levels in cultured media at 0, 24, and 48 h (*P* < 0.001; Figure 5). The amount of TAC was restored by cotreatment of *β*-cyfluthrin with 20 or 40 *μ*M luteolin at studied time points (*P* < 0.001; Figure 5). TAC levels were reduced at 48 h compared to 0 h in studied groups (*P* < 0.001; Figure 5).

#### 3.2.5. Intrahepatocyte Amount of TAC (mmol/g Protein)

**Figure 6:**
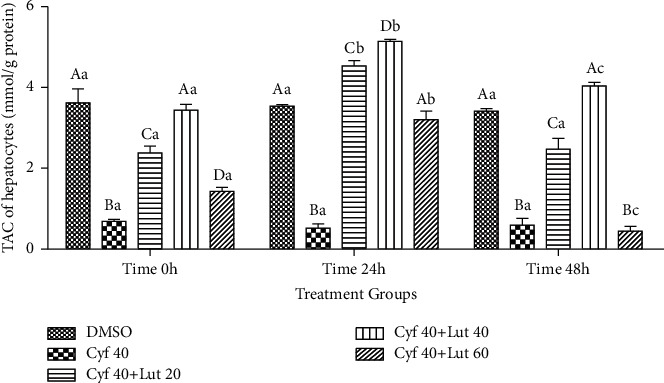
Intrahepatocyte total antioxidant capacity (TAC; mmol/g protein) level following exposure to toxic doses of *β*-cyfluthrin (40 *μ*M) alone or cotreatment with luteolin (20, 40, and 60 *μ*M). Hepatocytes were seeded at 2 × 10^5^ cells/well and were incubated for 48 h. Data are expressed as the mean ± SEM. ^A,B,C,D^Values with different letters indicate a difference (*P* < 0.001) among groups at each time point. ^a,b,c^Values with different letters indicate a difference (*P* < 0.001) over time within every experimental group. Cyf, *β*-cyfluthrin; Lut, luteolin; DMSO, dimethyl sulfoxide as a solvent of *β*-cyfluthrin and luteolin.

Within-group analysis revealed that the amount of TAC was lower in *β*-cyfluthrin alone, *β*-cyfluthrin + 20 *μ*M luteolin-treated, and *β*-cyfluthrin + 60 *μ*M luteolin-treated groups at 0 and 48 h compared to the relative control group (*P* < 0.001; Figure 6). Furthermore, a greater amount of TAC was recorded in *β*-cyfluthrin + 20 or 40 *μ*M luteolin-treated groups compared to the other treated groups at 48 h (*P* < 0.001; Figure 6). Over-time analysis indicated greater TAC values at 24 h compared to 0 h in luteolin-treated groups (*P* < 0.001; Figure 6).

#### 3.2.6. Amount of TNN (*μ*mol/g Protein) in the Cultured Media

**Figure 7:**
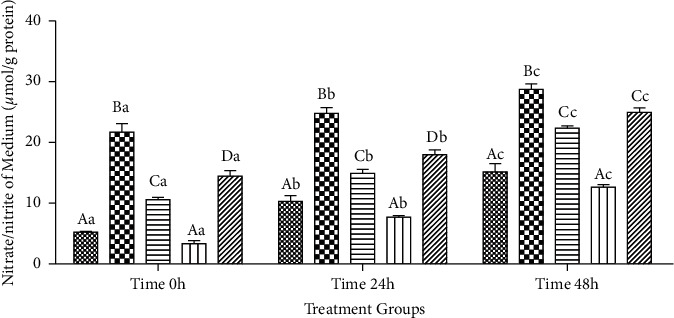
Total nitrate/nitrite (TNN; *μ*mol/g protein) in the medium of rat hepatocytes following exposure to toxic doses of *β*-cyfluthrin (40 *μ*M) alone or cotreatment with luteolin (20, 40, and 60 *μ*M). Hepatocytes were seeded at 2 × 10^5^ cells/well and were incubated for 48 h. Data are expressed as the mean ± SEM. ^A,B,C,D^Values with different letters indicate a significant difference (*P* < 0.05) among groups at each time point. ^a,b,c^Values with different letters indicate a difference (*P* < 0.05) over time within every experimental group. Cyf, *β*-cyfluthrin; Lut,luteolin; DMSO, dimethyl sulfoxide as a solvent of *β*-cyfluthrin and luteolin.

The time × treatment interaction revealed that the amount of TNN was greater in *β*-cyfluthrin-alone-treated group compared to other groups at 0, 24, and 48 h of storage (*P* < 0.001; Figure 7). Administration of luteolin at 40 *μ*M levels completely inhibits the deleterious effect of *β*-cyfluthrin. Within-group analysis indicated that the TNN amount increased in a time-dependent manner in all studied groups (*P* < 0.001; Figure 7).

#### 3.2.7. Intrahepatocyte Amount of TNN (*μ*mol/g Protein)

**Figure 8:**
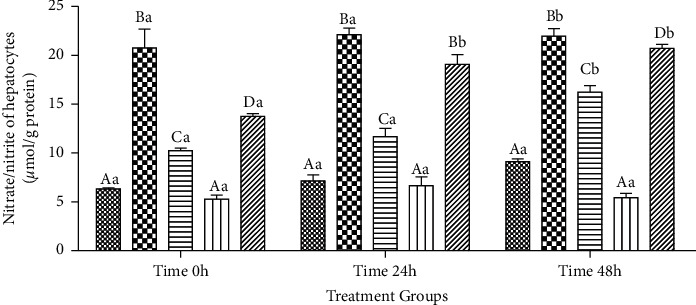
Intrahepatocyte total nitrate/nitrite (TNN; *μ*mol/g protein) following exposure to toxic doses of *β*-cyfluthrin (40 *μ*M) alone or cotreatment with luteolin (20, 40, and 60 *μ*M). Hepatocytes were seeded at 2 × 10^5^ cells/well and were incubated for 48 h. Data are expressed as the mean ± SEM. ^A,B,C,D^Values with different letters indicate a significant difference (*P* < 0.001) among groups at each time point. ^a,b^Values with different letters indicate a difference (*P* < 0.001) over time within every experimental group. Cyf, *β*-cyfluthrin; Lut, luteolin; DMSO, dimethyl sulfoxide as a solvent of *β*-cyfluthrin and luteolin.

The observed changes in the amount of intrahepatocyte TNN resembled that in the cultured media described above (Figure 8).

#### 3.2.8. Amount of TLHP (*μ*mol/g Protein) in the Cultured Media

**Figure 9:**
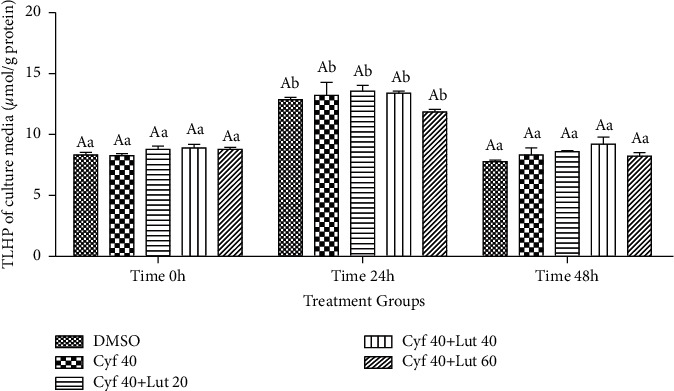
Amount of total lipid hydroperoxide (TLHP; *μ*mol/g protein) in the cultured medium of rat hepatocytes following exposure to toxic doses of *β*-cyfluthrin (40 *μ*M) alone or cotreatment with luteolin (20, 40, and 60 *μ*M). Hepatocytes were seeded at 2 × 10^5^ cells/well and were incubated for 48 h. Data are expressed as the mean ± SEM. Significant changes were not observed among groups at each time point (*P* > 0.05). ^a,b^Values with different letters indicate a difference (*P* < 0.05) over time within every experimental group. Cyf, *β*-cyfluthrin; Lut, luteolin; DMSO, dimethyl sulfoxide as a solvent of *β*-cyfluthrin and luteolin.

There were no significant changes among treated groups at different time points (*P* > 0.05; Figure 9). Within-group analysis indicated a greater amount of TLHP at 24 h compared to 0 and 48 h time points in all groups (*P* < 0.05; Figure 9).

#### 3.2.9. Intrahepatocyte Amount of TLHP (*μ*mol/g Protein)

**Figure 10:**
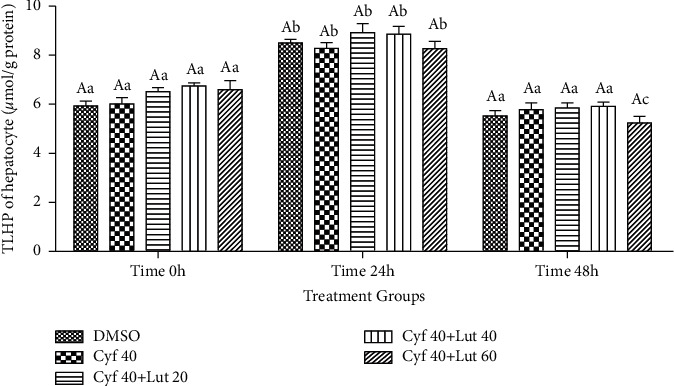
Intrahepatocyte total lipid hydroperoxide (TLHP; *μ*mol/g protein) level following exposure to toxic doses of *β*-cyfluthrin (40 *μ*M) alone or cotreatment with luteolin (20, 40, and 60 *μ*M). Hepatocytes were seeded at 2 × 10^5^ cells/well and were incubated for 48 h. Data are expressed as the mean ± SEM. Significant changes were not observed among groups at each time point (*P*=0.102). ^a,b,c^Values with different letters indicate a difference (*P* < 0.05) over time within experimental groups. Cyf, *β*-cyfluthrin; Lut, luteolin; DMSO, dimethyl sulfoxide as a solvent of *β*-cyfluthrin and luteolin.

Changes in TLHP within hepatocytes were the same as that in the culture media (Figure 10).

#### 3.2.10. Activity of SOD in the Culture Media (unit/mg Protein)

**Figure 11:**
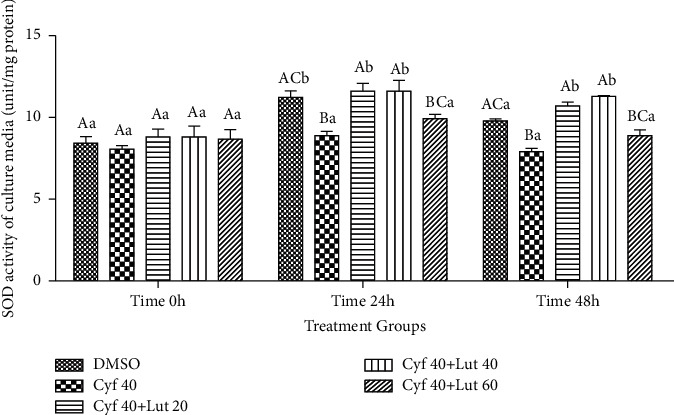
Superoxide dismutase (SOD; unit/mg protein) activity in the cultured medium of rat hepatocytes following exposure to toxic doses of *β*-cyfluthrin (40 *μ*M) alone or cotreatment with luteolin (20, 40, and 60 *μ*M). Hepatocytes were seeded at 2 × 10^5^ cells/well and were incubated for 48 h. Data are expressed as the mean ± SEM. ^A,B,C^Values with different letters indicate a significant difference (*P* < 0.001) among groups at each time point. ^a,b^Values with different letters indicate a difference (*P* < 0.001) over time within every experimental group. Cyf, *β*-cyfluthrin; Lut, luteolin; DMSO, dimethyl sulfoxide as a solvent of *β*-cyfluthrin and luteolin.

The activity of SOD was greater in control and *β*-cyfluthrin + 20 or 40 *μ*M luteolin groups compared to the *β*-cyfluthrin-only-exposed group at 24 and 48 h (*P* < 0.01; Figure 11). SOD activity of *β*-cyfluthrin + 20 or 40 *μ*M luteolin groups was higher at 24 and 48 h compared to 0 h (*P* < 0.01; Figure 11).

#### 3.2.11. The Activity of SOD within Hepatocytes (unit/mg Protein)

**Figure 12:**
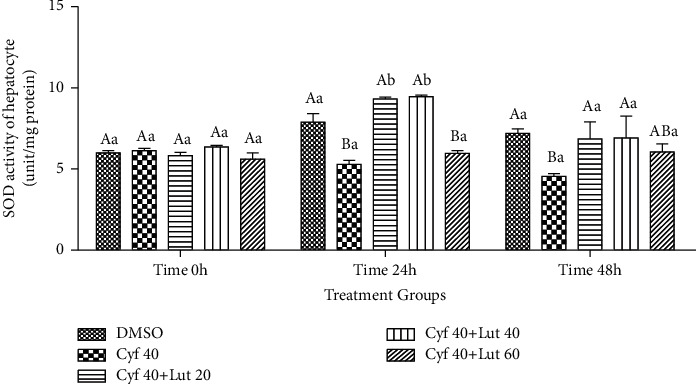
Intrahepatocyte SOD activity (unit/mg protein) in the medium of rat hepatocytes following exposure to toxic doses of *β*-cyfluthrin (40 *μ*M) alone or cotreatment with luteolin (20, 40, and 60 *μ*M). Hepatocytes were seeded at 2 × 10^5^ cells/well and were incubated for 48 h. Data are expressed as the mean ± SEM. ^A,B^Values with different letters indicate a significant difference (*P* < 0.001) among groups at each time point. ^a,b^Values with different letters indicate a difference (*P* < 0.001) over time within every experimental group. Cyf, *β*-cyfluthrin; Lut, luteolin; DMSO, dimethyl sulfoxide as a solvent of *β*-cyfluthrin and luteolin.

The activity of SOD was lower in *β*-cyfluthrin-only-treated group than the control group at 24 and 48 h of storages (*P* < 0.01; Figure 12). Coadministration of 20 or 40 *μ*M luteolin restored the activity of the mentioned enzyme within hepatocytes compared to the *β*-cyfluthrin-alone-treated group. Within-group analysis revealed greater activity at 24 h than 0- and 48-h time points in all groups (*P* < 0.01; Figure 12).

#### 3.2.12. Correlation between Functionality of Hepatocytes with Other Variables

The analysis revealed that there was a positive correlation between MTT and TAC (*P* < 0.001; Supplementary file 1) and SOD (*P*=0.003; Supplementary file 1) and negative correlation with MDA (*P* < 0.001; Supplementary file 1) and TNN (*P* < 0.001; Supplementary file 1). There was no association between MTT and TLHP variables (*P*=0.48; Supplementary file 1).

## 4. Discussion

The first aim of the present study was to assess the varying amounts of *β*-cyfluthrin on the viability (evaluated by MTT) of cultured rat primary hepatocytes up to 48 h. The analysis of data revealed that *β*-cyfluthrin at 40 and 80 *μ*M reduced the viability of hepatocytes at 24 and 48 h after exposure; therefore, the dose of 40 *μ*M was selected and used in the following experiment. Another objective was to assess the capability of luteolin, as an antioxidant, in the alleviation of *β*-cyfluthrin-induced hepatotoxic effects.

Research indicated that pyrethroids induce oxidative toxicity when exposed to humans, mice, or rats during *in vivo* and *in vitro* experiments [33–37]. Due to the presence of the cyano group in the structure of *β*-cyfluthrin, its toxicity might be related to the release of chemically unstable cyanohydrins after exposure [38]. In biological systems, cyanohydrins change to cyanides and aldehydes, which ultimately could act as free radicals [12]. Moreover, due to the high lipophilicity of *β*-cyfluthrin, it could easily penetrate via cell membranes and exert its detrimental effects within the cells [39]. Previous reports revealed that peroxidative reaction is one of the pathways of induced toxicity by pyrethroid insecticides over *in vitro *exposure [10, 40, 41]. According to the results of MTT (in the first and second experiments) and MDA levels, the current study confirmed that *β*-cyfluthrin induces oxidative toxicity in the primary culture of rat hepatocytes. Moreover, the results of the current study indicated the protective role of luteolin against the toxic effects of *β*-cyfluthrin on viability and MDA levels of rat hepatocytes. Previous research revealed that luteolin, which belongs to a class of flavonoids, has strong ROS scavenger effects [18, 42]. In this regard, it has been demonstrated that the glycosylated form of luteolin (cynaroside) reduced the amount of ROS generation and ultimately prevented the deleterious effects of oxidative toxicity in cardiomyocytes [43]. Another experiment indicated the positive effects of luteolin in a dose-dependent (30–100 *μ*M) manner on free radical-scavenging activity and index of lipid peroxidation levels during induced oxidative toxicity in the mouse brain cells [44]. Moreover, the protective role of luteolin against detrimental effects of isoproterenol-induced myocardial infarction on different indices, such as MDA and lipid hydroperoxides, was demonstrated in treated male Wistar rats [45]. Our findings are in good accordance with the previous reports about the role of luteolin in increasing the viability of cells when exposed to oxidant compounds such as cisplatin and H_2_O_2_ [41, 42].

Our results indicated that *β*-cyfluthrin at 40 and 80 *μ*M levels reduced the viability of rat hepatocytes during primary culture. In accordance with our results, degenerative changes and reduced hepatocyte viability were indicated after exposure to cypermethrin or pyrrolidine alkaloids [46, 47]. The reduction of hepatocyte viability in the group treated with *β*-cyfluthrin (alone) might be strongly related to the generation of free radicals, stimulation of peroxidative and nitrosative reactions, a significant increase in MDA and TNN levels, and ultimately reduction of enzymatic antioxidant amount [10, 48]. Moreover, the results of the current study revealed the hepatoprotective role of luteolin against toxic doses of *β*-cyfluthrin. In this regard, increased cell viability following treatment with luteolin, especially during cell challenges with oxidants, was attributed to enhancement of antiapoptotic protein Bcl-2 expression and lowering the expression of the proapoptotic protein Bax in a dose-dependent manner [49].

Enzymatic antioxidants act effectively to reduce the amounts of ROS and ultimately their detrimental oxidative effect on biological systems [35]. It seems that a slight reduction in the physiological amounts of enzymatic antioxidants, such as SOD, reduces the capability and the resistance of cellular lipids, proteins, and DNA to counteract against detrimental effects of oxidative toxicity [50, 51]. Jebur et al. [35] indicated the reduction of hepatic enzymatic and nonenzymatic levels of antioxidants (reduced glutathione, glutathione peroxidase, glutathione reductase, glutathione S-transferase, SOD, and catalase) when rats were exposed to 15 mg/kg BW of *β*-cyfluthrin via oral gavage. Results of the current study displayed that *β*-cyfluthrin significantly reduces the amount of SOD activity within both hepatocytes and the cultured media. It is hypothesized that *β*-cyfluthrin directly (inhibition of SOD activity) or indirectly (consumption during neutralization of free radicals) caused a reduction in enzymatic antioxidants [35]. Our findings indicated the ameliorative role of luteolin against the detrimental effects of *β*-cyfluthrin on TAC and SOD levels. In accordance with our findings, the protective role of luteolin (10 and 20 *μ*g/mL) on hemolysis rate, ROS generation, MDA, and amounts of antioxidative enzymes when erythrocytes were treated with the known oxidant compound (H_2_O_2_) was shown [52]. Luteolin was significantly effective in alleviating the detrimental effects of isoproterenol-induced myocardial infarction on different indices, such as MDA, lipid hydroperoxides, and enzymatic and nonenzymatic antioxidant levels of treated male Wistar rats [45]. The cytoprotective effect of luteolin due to its strong antioxidative properties and scavenging of ROS has been reported in the cell culture model [53]. Our results exhibited that luteolin would reduce the levels of nitrosative reaction, as measured by total nitrate-nitrite, mediated by *β*-cyfluthrin. In this regard, the modulatory role of luteolin on oxidative and nitrosative indices of mouse retinal cells was reported [54]. The extent of cell damage by oxidants is reduced by enhancing heme oxygenase-1 (HO-1) expression [55]. The mentioned enzyme (act as antioxidative/cytoprotective) is usually induced by exposure to oxidants such as UV irradiation, proinflammatory cytokines, heavy metals, and thiol-reactive substances [56]. However, the expression of HO-1 was not evaluated in the current study, but the direct role of luteolin on the augmentation of TAC levels through HO-1 expression was shown previously [41]. In accordance with our results, Sun et al. [49] reported that luteolin showed the capability to ameliorate the harmful effects of isoproterenol on TAC and SOD activities during cell culture experiments. Additionally, they revealed that luteolin reduced the MDA and ROS levels during H_2_O_2_-mediated toxicity in a dose-dependent manner, similar to our findings. Based on the results of the previous [15–24] and current studies about a wide range of pharmacological effects of the luteolin, it is recommended that the consumption of foods containing luteolin prevents or reduces the adverse effects of diseases or oxidative stress.

The current experiment displayed that the most protective effect of luteolin was achieved at the doses of 20 and 40 *μ*M but reduced at 80 *μ*M. There is increasing evidence showing that the natural substances with antioxidant effects are acting as double-edged swords, meaning that the high concentration of exogenous antioxidants may disrupt redox balance. It seems that the higher concentrations of exogenous antioxidant act like a pro-oxidant and activates pathways such as increasing proinflammatory mediators' production, nitrosylation of proteins, proglycation effect, and endocrine-disrupting activities [57]. Therefore, the results of the present experiment concerning the reverse effect of the luteolin supplementation at 80 *μ*M level might be related to nonphysiologic concentration of exogenous antioxidant, which was observed in the current experiment.

In conclusion, exposure of rat hepatocytes to toxic doses of *β*-cyfluthrin reduced the SOD activity and TAC levels and increased the nitrosative and peroxidative indices. Furthermore, the capability of luteolin to alleviate the detrimental effects of *β*-cyfluthrin on the primary culture of rat hepatocytes was indicated by the current study. However, more studies are mandatory to indicate the protective role of luteolin upon induced toxicity of hepatocytes (by toxicants such as *β*-cyfluthrin) at the gene level.

## Data Availability

The data used to support the findings of this study are available from the corresponding author upon request.

## Supplementary Materials

Supplementary Materials Supplementary file 1: the correlation and regression between MTT (% of functional hepatocytes) and MDA, TAC, TNN, SOD, and TLHPs of hepatocytes.
