# Supplementary material for: β-Cyfluthrin-Mediated Cytotoxicity of Cultured Rat Primary Hepatocytes Ameliorated by Cotreatment with Luteolin
Source: Evid Based Complement Alternat Med. 2022 Aug 27;2022:3647988. doi: 10.1155/2022/3647988 (PMC9440783; doi:10.1155/2022/3647988)
Supplement: Supplementary Materials — Supplementary file 1: the correlation and regression between MTT (% of functional hepatocytes) and MDA, TAC, TNN, SOD, and TLHPs of hepatocytes. [file 3647988.f1.docx]

Supplementary file 1. The correlation and regression between MTT (% of functional hepatocytes) with MDA, TAC, TNN, SOD, and TLHP amounts of hepatocytes.
